# Supplementary material for: Relative Telomere Length Change in Colorectal Carcinoma and its Association with Tumor Characteristics, Gene Expression and Microsatellite Instability
Source: Cancers (Basel). 2022 Apr 30;14(9):2250. doi: 10.3390/cancers14092250 (PMC9105685; doi:10.3390/cancers14092250)
Supplement: Supplementary file 1 [file cancers-14-02250-s001.zip › cancers-1689374-final-suppl method.pdf]

Supplemental Method:

Assay for Relative Telomere Length (RTL) measurement:

The Luminex assay is a probe-based assay, where we use oligonucleotide probes for Telomeric repeat region (TEL) and a probe for a reference gene *ALK*. The quantification of TEL and *ALK* probe is done against a standard curve generated from a standard DNA sample. RTL is expressed as the ratio of TEL/*ALK*. Therefore, it is normalized for quantity of DNA in the same well, and it is relative to the "standard" DNA sample [17].

From this point of view, this is a similar approach like qPCR measurement where Telomere is compared to a reference gene [37,38]. The major difference is, the Luminex assay does not require amplification of the DNA sample making it free of amplification bias. In addition, the reproducibility is very good without any well-to-well effect that is seen in qPCR [39].

In this high-throughput Luminex assay [17], four 96-well plates are processed simultaneously upto the hybridization step, each plate containing 60 test DNA samples (the remaining wells are used for standard and controls), and the corresponding wells of four such plates were pooled in a single plate for all the subsequent steps: signal amplification, attaching detection dye, wash and final detection of signal intensity. Tumor and corresponding normal samples from same patient were placed in the same plate.

The Luminex assay does not provide the absolute telomere length (in base pair). The measurement of absolute telomere length (e.g. Southern blot) may be better, but requires a much larger quantity of DNA (at least 2-3 microgram compared to only 50 ng in Luminex assay) [40,41] and more importantly is many folds more expensive and time consuming which restricts the use of Southern blot in large-scale studies.

Reproducibility of the assay: In a recent study, RTL was measured for 3123 tissue samples (up to 12 tissue types per donor) using this high throughput Luminex assay [42]. In that study, as a part of QC, a total of 233 samples were randomly duplicated and the laboratory was totally blinded to their identities. There was no difference between the two measurements (1.11 6SD 0.348 vs. 1.10 6SD 0.356, paired t-test P=0.614). The ICC between these two measurements using an absolute agreement definition was 0.897 (95% CI 0.866–0.920).

Validation of the Luminex assay against gold standard: The Luminex assay for telomere correlates well with the traditional gold standard TRF Southern blot. In a blinded comparison, the Luminex and Southern blot measurements for the same 50 DNA samples were taken in two independent laboratories; each sample was measured twice, several months apart. The correlation (r) between Southern blot and Luminex was 0.65 in round-1 and 0.75 in round-2 [18].

External validation of the Luminex assay: Telomere length is known to have a negative correlation with age. Such a known negative correlate may be used as external validity of an assay [43,44]. In an independent set of DNA samples from 505 individuals (m=329, f=176), the leukocyte RTL was measured by this assay. The subjects were between 21 years and 70 years of age. Linear regression analysis suggested that, with an increase of age by 10 years, the RTL decreased by 0.08 (95% CI 0.09-0.06) [17]. The RTL negatively correlated with age (r=-0.41, P<.0001). In other words, 17.5% of variation (r^2=0.175) in RTL was explained by variation in age. In a previous study using the original lower throughput Luminex assay, it was found that 14.5% of variation (r^2=0.14) in RTL was explained by variation in age [18].

Genome-wide Gene Expression assay:

We used microarray data (Illumina HT12 v4 BeadChip) from the first 71 paired tumor and normal tissue RNA (of the same set of 165 patients used for RTL assay in this study). The chip contains a total of 47,231 probes covering 31,335 genes. Paired samples were processed in same chip (12 samples/ chip). One sample from normal tissue failed on the microarray. So, we had gene expression data from 71 CRC tissue and 70 corresponding normal tissue. Gene expression data was normalized using quantile normalization in GenomeStudio software.

Genome-wide methylation assay

We also have methylation data (Illumina HumanMethylation450 DNA analysis BeadChip v1.0 Assay) from the first 125 paired tumor-normal samples (125 pair out of the same set of 165 patients used for this study) [45]. The DNA samples were subjected to bisulfite conversion using EZ-96 DNA Methylation Kit (Zymo Research, USA).The chip presents 485,577 loci of which 150,254 in CpG Island, 112,067 in Shore (0-2kb from island), 47,114 in Shelf (2-4kb from the island) and 176,112 in deep sea (>4kb from CpG island). Paired samples (CRC and corresponding normal) were processed on the same chip to avoid batch effect. From this assay on average 17 loci per gene were interrogated. A Tecan Evo robot was used for automated sample processing and the chips were scanned on a single iScan reader. If the intensity of Methylated loci is X and the intensity of unmethylated loci is Y, then, Methylation score (beta value) is X/X+Y. If all are unmethylated (X=0), then methylation level is 0/0+Y= 0. If all loci are methylated (Y=0), then beta value is X/X+0=

1. If 50% probes are hybridized at methylated loci and 50% hybridized at unmethylated loci then methylation score is 50/50+50= 0.5.

Microsatellite Instability (MSI) detection

A high-resolution melting (HRM) analysis method was used for detection of two mononucleotide MSI markers – BAT25 and BAT26 [19,46]. A tumor was defined as having MSI when it showed instability with at least one of these markers (BAT25 and BAT26), and as MSS when it showed no instability for both the markers. We used published primer sequences [46]. The amplification conditions included the polymerase activation step at 95 °C for 2 min, followed by 5 cycles of denaturation at 95 °C for 15 sec, annealing starting at 60 °C for 30 sec, extension at 72 °C for 30 sec, and an additional 33 cycles of denaturation at 95 °C for 15 sec, annealing at 53 °C for 30 sec, and extension at 72 °C for 30 sec. Before the HRM step, the products were heated to 95 °C for 1 min and cooled to 40 °C for 1 min, to allow heteroduplex formation. HRM was carried out and the data collected over the range from 60 to 95 °C, with temperature increment of 0.2°C/sec at each 0.05 sec. The BAT25 and BAT 26 products were sequenced for validation. In this way, a total of 30 tumor samples showed MSI and all were confirmed by another relatively novel MSI marker CAT25 [47,48].

KRAS and BRAF mutation detection:

Tumor and adjacent healthy colonic tissue from 165 paired (tumor and normal) tissue were tested for KRAS (rs 112445441) and BRAFV600E mutation by High Resolution Melt analysis as described previously [19].

Statistical Analysis:

To compare the continuous variables, we used t-test or one-way analysis of variance (ANOVA). Principal component analysis (PCA) and sample histograms were checked as a part of quality control analyses of the microarray data. Mixed-model multi-way ANOVA (which allows more than one ANOVA factor to be entered in each model) was used to compare the individual probe level expression data (for gene expression) or beta value of CpG loci (for methylation data) across different groups. In general, "tissue" (tumor/adjacent normal), telomere shortening (0= no, 1= yes), MSI status (MSI/MSS) were used as categorical variables with fixed effect since the levels "tumor/normal", "telomere shortening yes/no", "MSI/MSS" represent all conditions of interest; whereas "person ID#" (as proxy of inter-person variation) was treated as categorical variable with random effect, since the person ID is only a random sample of all the levels of that factor. Method of moments estimation was used to obtain estimates of variance components for mixed models [49]. As per the study design, we processed both the CRC tissue and the corresponding adjacent normal sample from one individual in a single chip. In the ANOVA model, the $\log_2$-transformed gene expression or beta-value for the CpG loci was used as the response variable (Y), and "Tumor" (tumor or normal), person ID#, "MSI-status" and "telomere shortening" were entered as ANOVA factors.

For paired analysis, we used the following model:

$$Y_{ijk} = \mu + Tumor_i + Person_j + \varepsilon_{ijk}$$

Where Y*ijk* represents the *k-th* observation on the *i-th* Tumor *j-th* Person. μ is the common effect for the whole experiment. εijk represents the random error present in the *k-th* observation on the *i-th* Tumor *j-th* Person. The errors εijk are assumed to be normally and independently distributed with mean 0 and standard deviation δ for all measurements. Person is a random effect.

For detection of interaction between Tumor and Telomere shortening, the following model was used:

$$Y_{ijk} = \mu + Tumor_i + Telomere\ shortening_j + Tumor * Telomere\ shortening_{ij} + \varepsilon_{ijk}$$

Where Y*ijk* represents the *k-th* observation on the *i-th* Tumor *j-th* Telomere shortening. μ is the common effect for the whole experiment. εijk represents the random error present in the *k-th* observation on the *i-th* Tumor *j-th* Telomere shortening. The errors εijk are assumed to be normally and independently distributed with mean 0 and standard deviation δ for all measurements.

Gene Ontology (GO) was used to group a set of genes into a category. In GO Enrichment analysis, we tested if the genes found to be differentially expressed or methylated fell into a Gene Ontology category more often than expected by chance [50]. We used chi-square test to compare "number of significant genes from a given category/total number of significant genes" vs. "number of genes on chip in that category/total number of genes on the microarray chip". Negative log of the p-value for this test was used as the enrichment score. Therefore, a GO group with a high enrichment score represents a lead functional group. The enrichment scores were analyzed in a hierarchical visualization and in tabular form.

Gene set ANOVA is a mixed model ANOVA to test the expression or methylation of a set of genes (sharing the same category or functional group) instead of an individual gene in different groups [50]. The analysis is performed at

the gene level, but the result is expressed at the level of the GO-category by averaging the member genes' results. The equation for the model was:

$$\text{Model}: Y = \mu + T + P + G + S\,(T * P) + \varepsilon$$

where Y represents the expression or methylation status of a Gene set category, μ is the common effect or average expression / methylation of the Gene set category, T is the tissue-to-tissue (tumor/ normal) effect, P is the patient-to-patient effect, G is the gene-to-gene effect (differential expression or methylation of genes within the gene set category independent of tissue types), S (T*P) is the sample-to-sample effect (this is a random effect, and nested in tissue and patient) and ε represents the random error.
