# Supplementary figures and images for: Relative Telomere Length Change in Colorectal Carcinoma and its Association with Tumor Characteristics, Gene Expression and Microsatellite Instability
Source: Cancers (Basel). 2022 Apr 30;14(9):2250. doi: 10.3390/cancers14092250 (PMC9105685; doi:10.3390/cancers14092250)

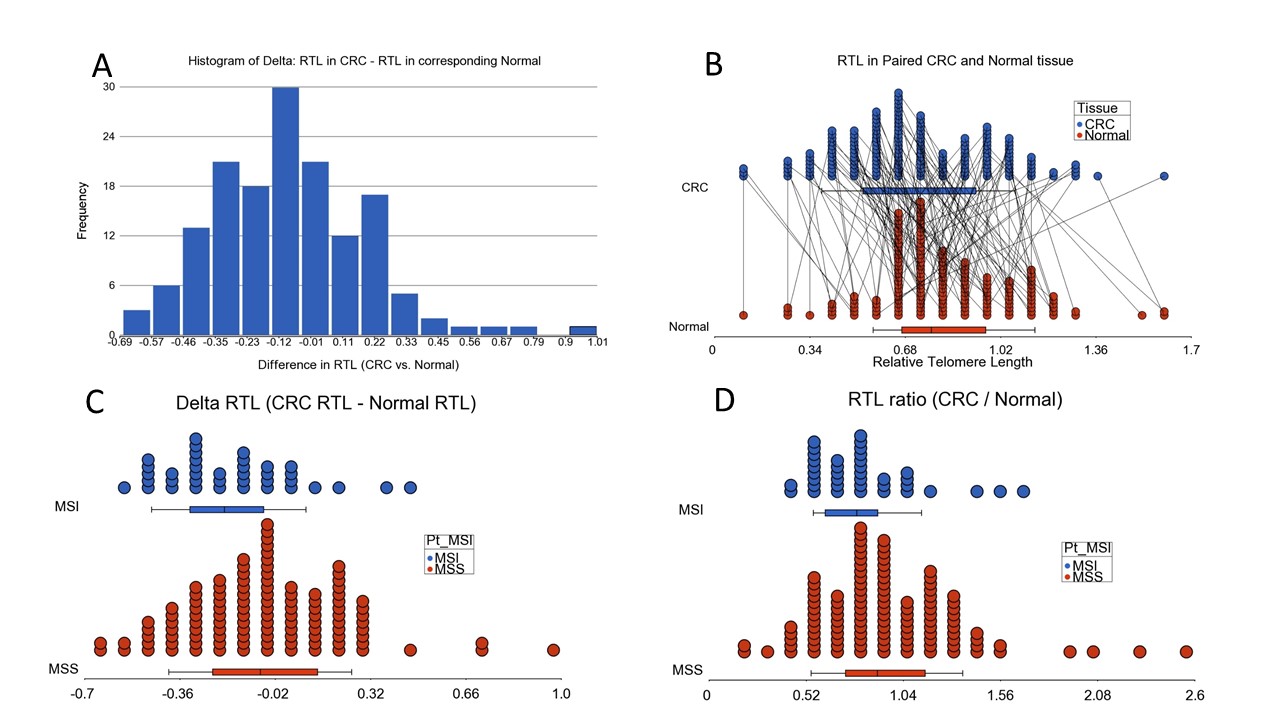

Supplement: Supplementary file 1 [file cancers-14-02250-s001.zip › Figure S1.jpg]

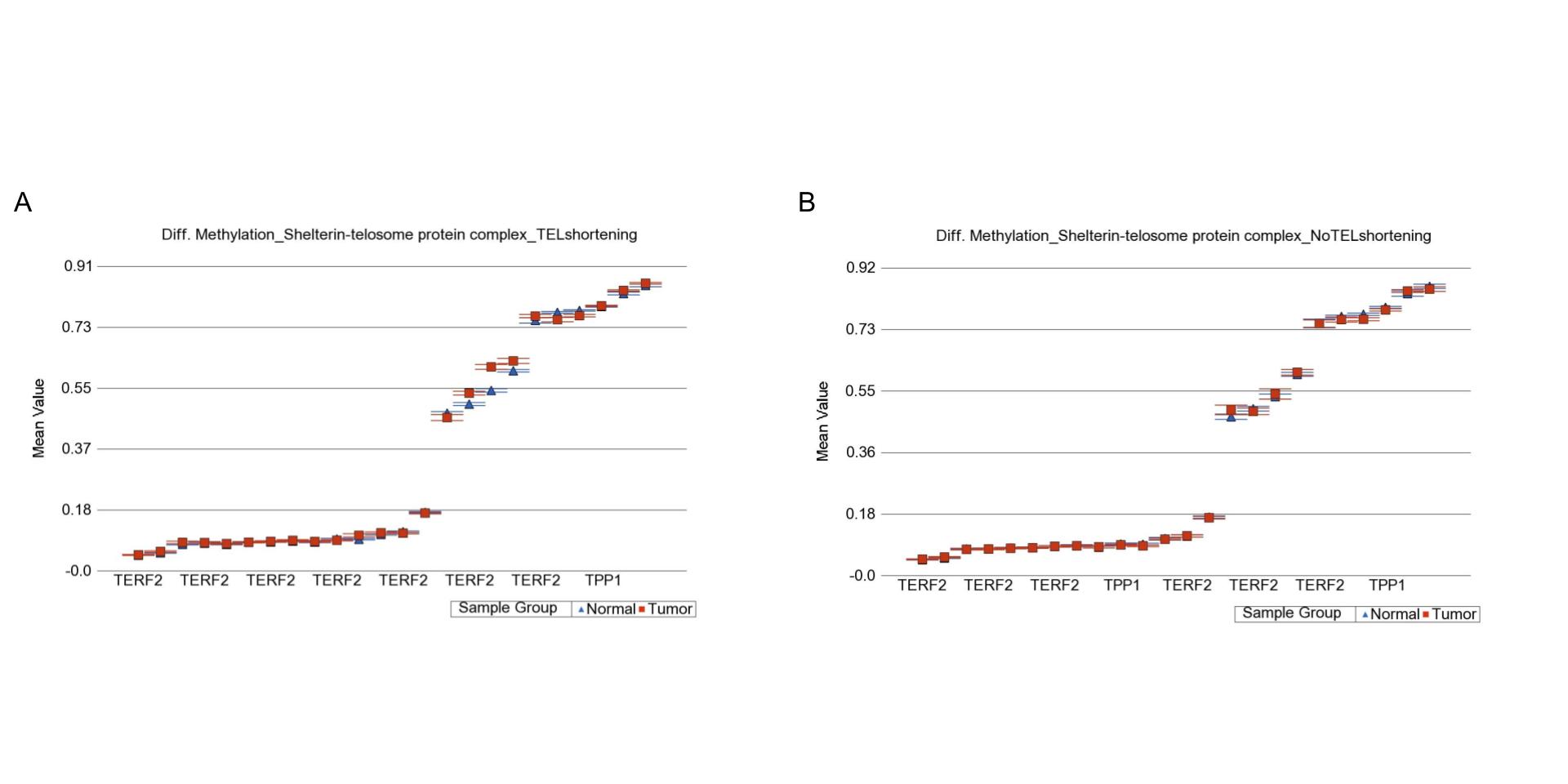

Supplement: Supplementary file 1 [file cancers-14-02250-s001.zip › Figure S10.jpg]

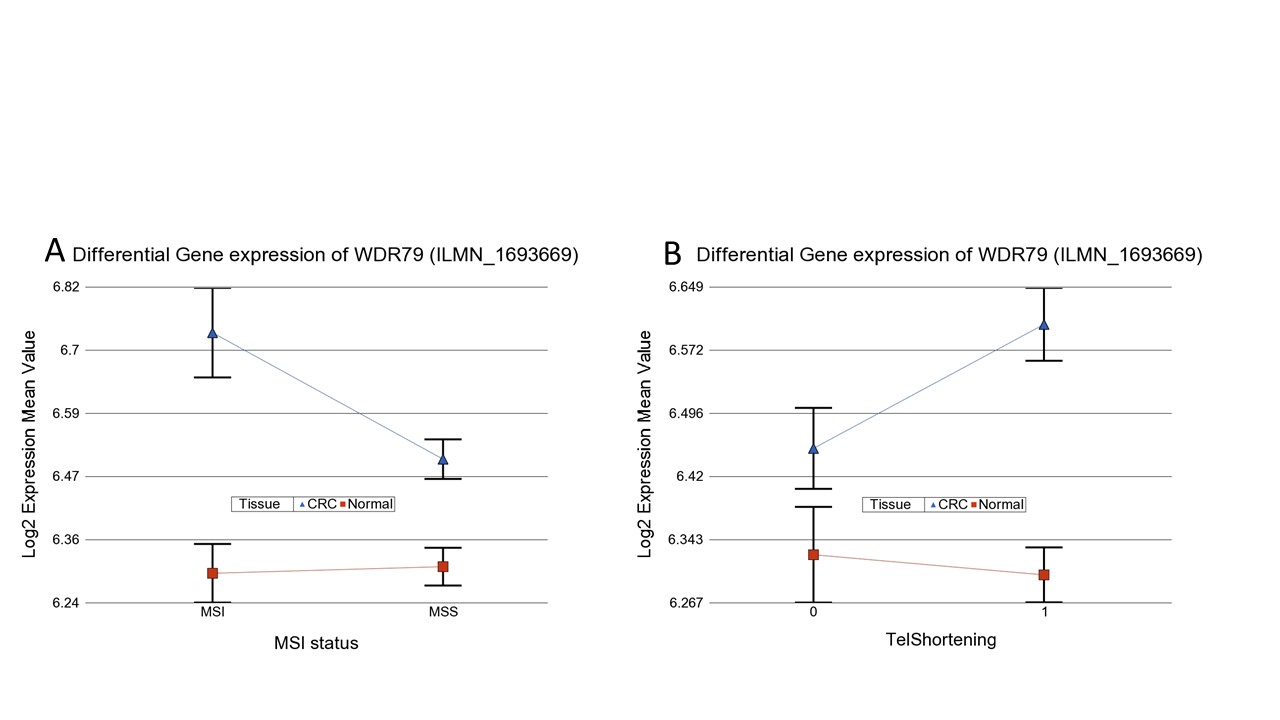

Supplement: Supplementary file 1 [file cancers-14-02250-s001.zip › Figure S11.jpg]

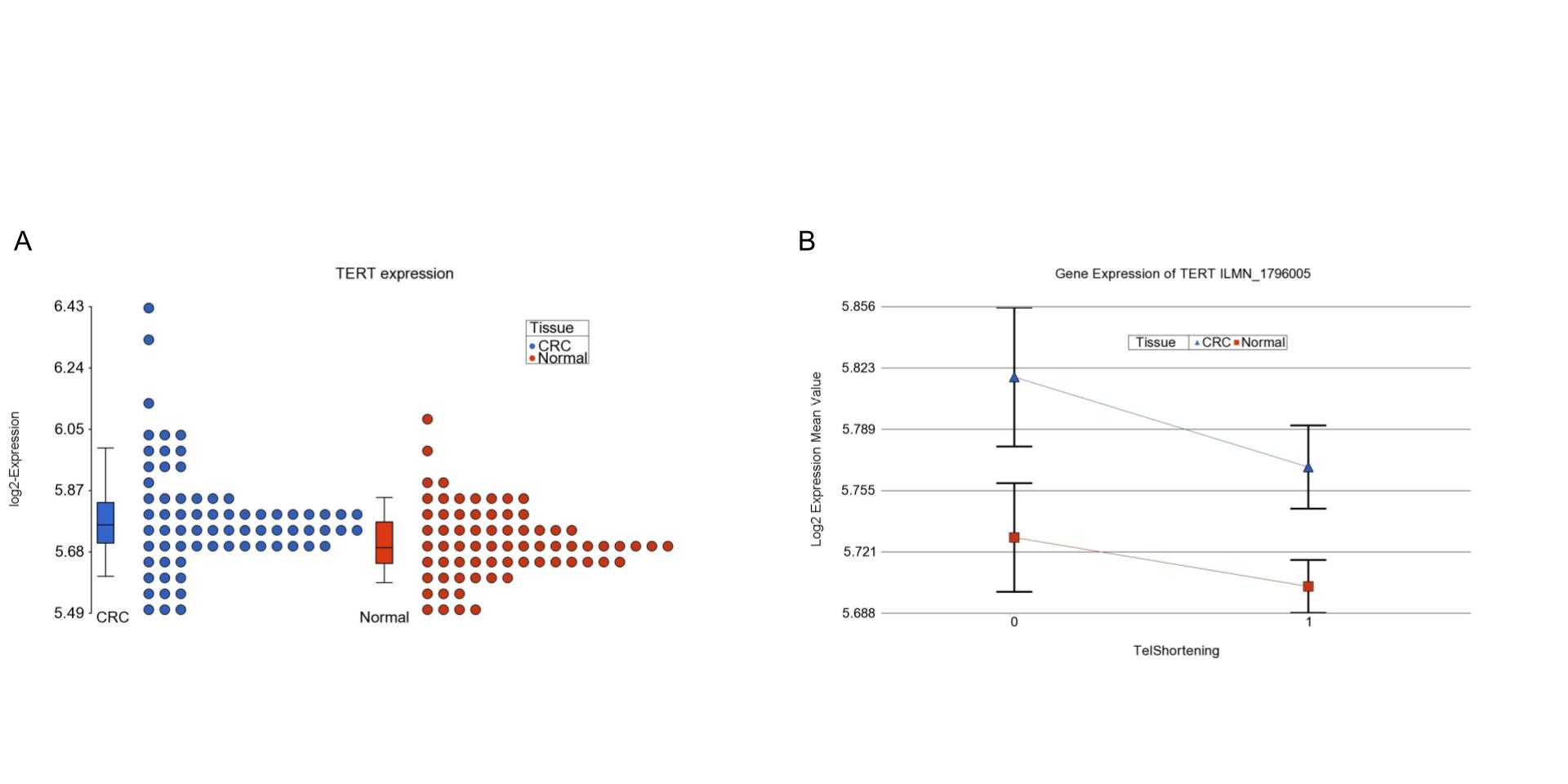

Supplement: Supplementary file 1 [file cancers-14-02250-s001.zip › Figure S2.jpg]

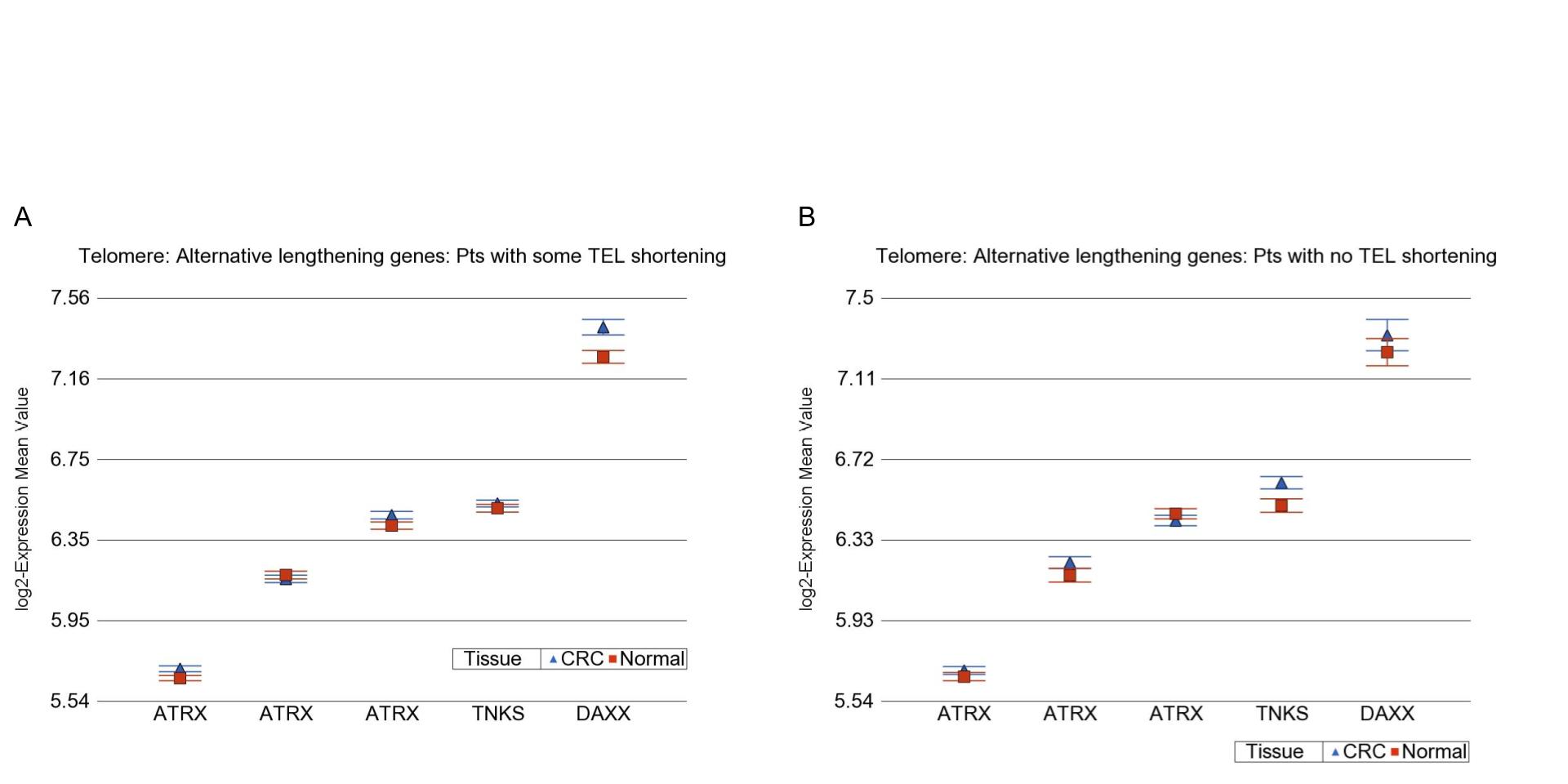

Supplement: Supplementary file 1 [file cancers-14-02250-s001.zip › Figure S3.jpg]

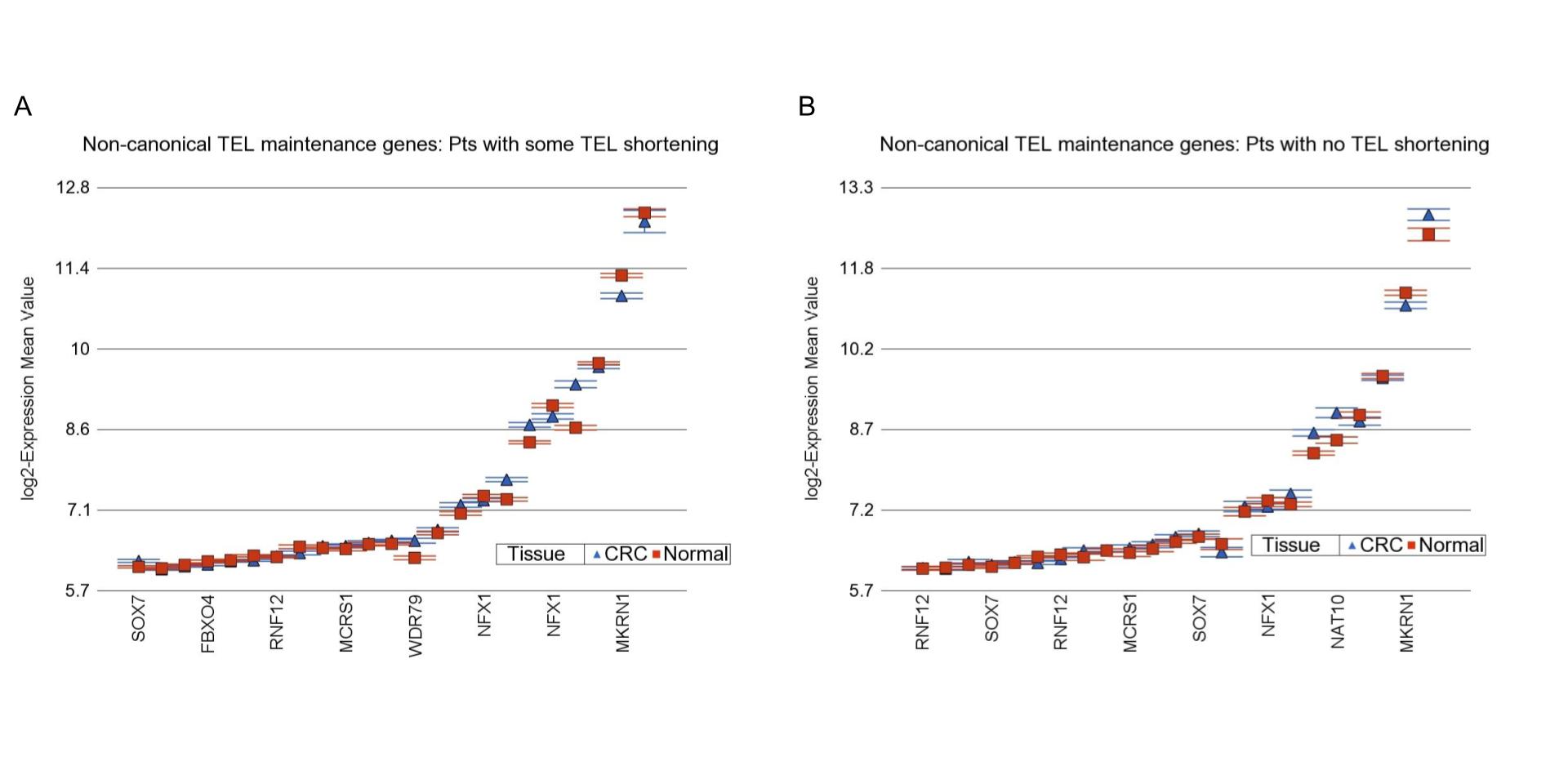

Supplement: Supplementary file 1 [file cancers-14-02250-s001.zip › Figure S4.jpg]

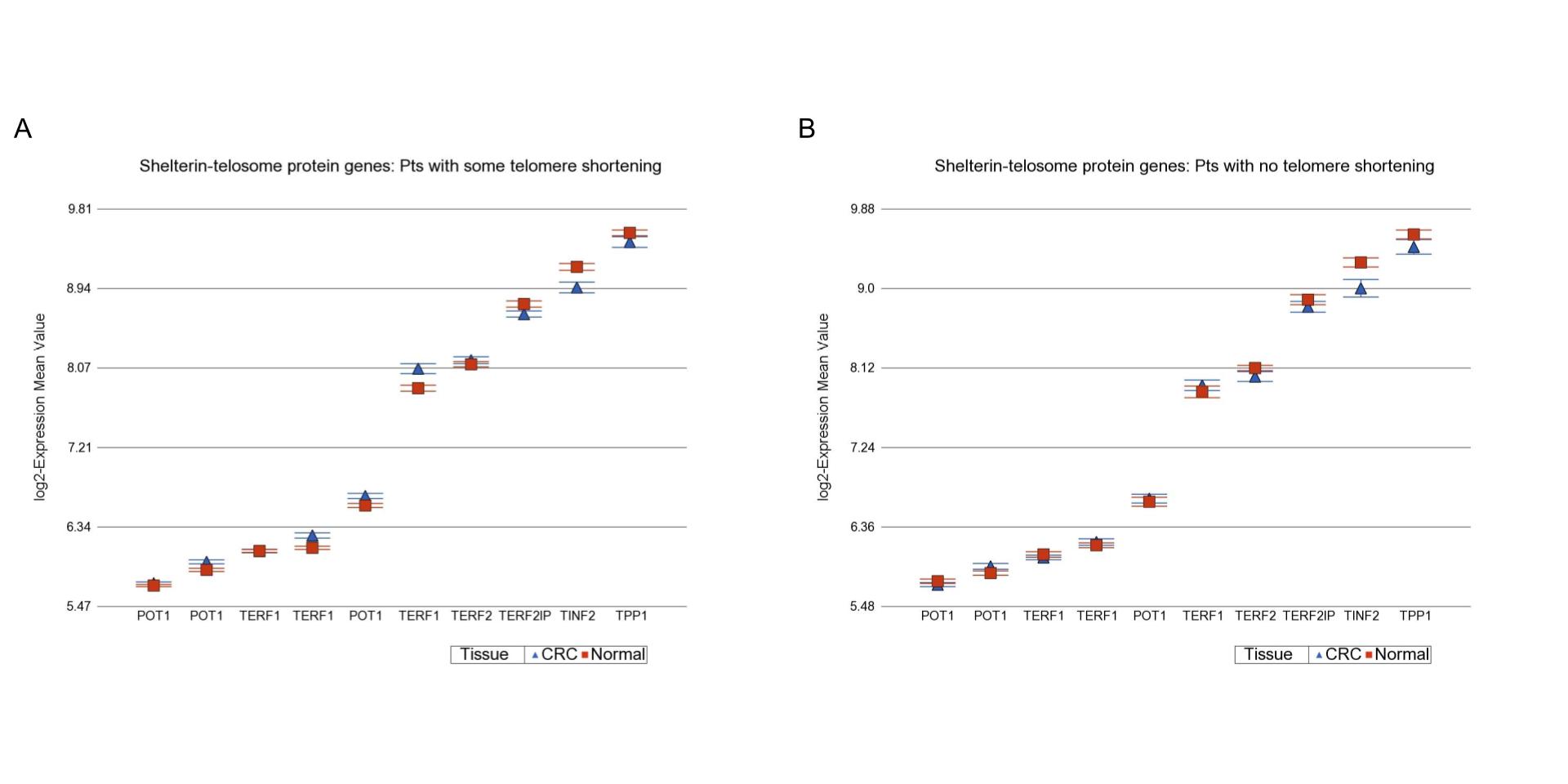

Supplement: Supplementary file 1 [file cancers-14-02250-s001.zip › Figure S5.jpg]

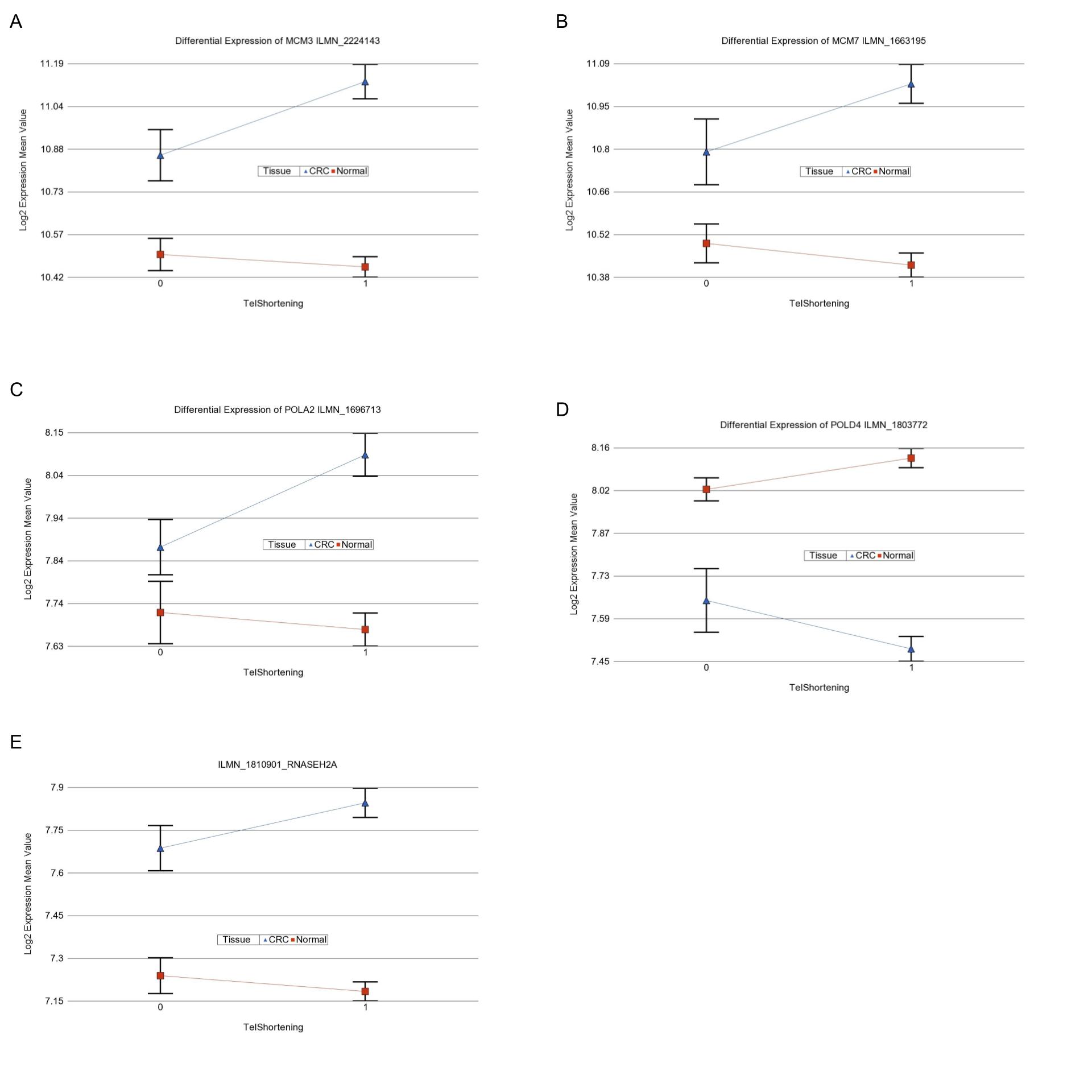

Supplement: Supplementary file 1 [file cancers-14-02250-s001.zip › Figure S6.jpg]

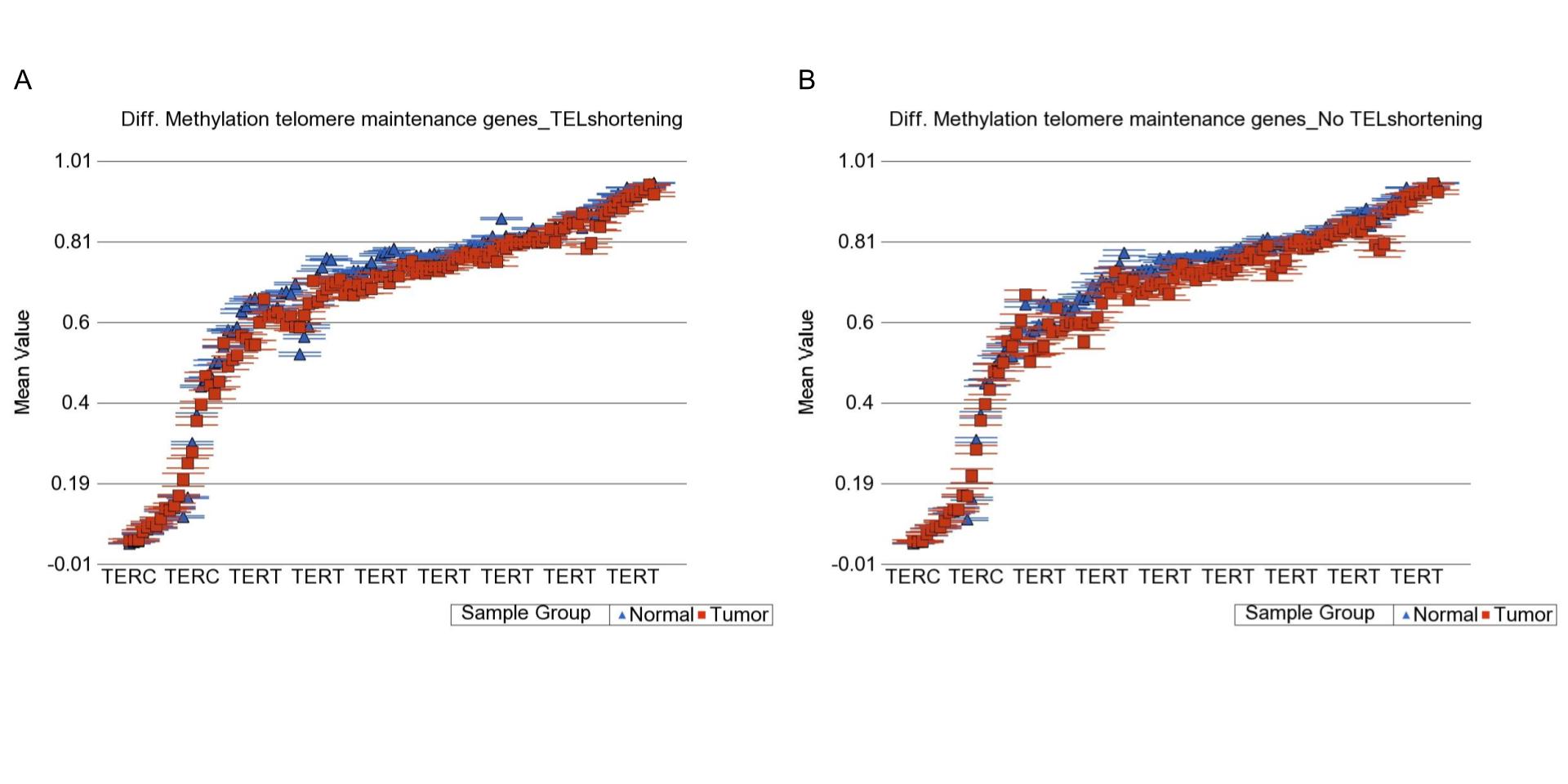

Supplement: Supplementary file 1 [file cancers-14-02250-s001.zip › Figure S7.jpg]

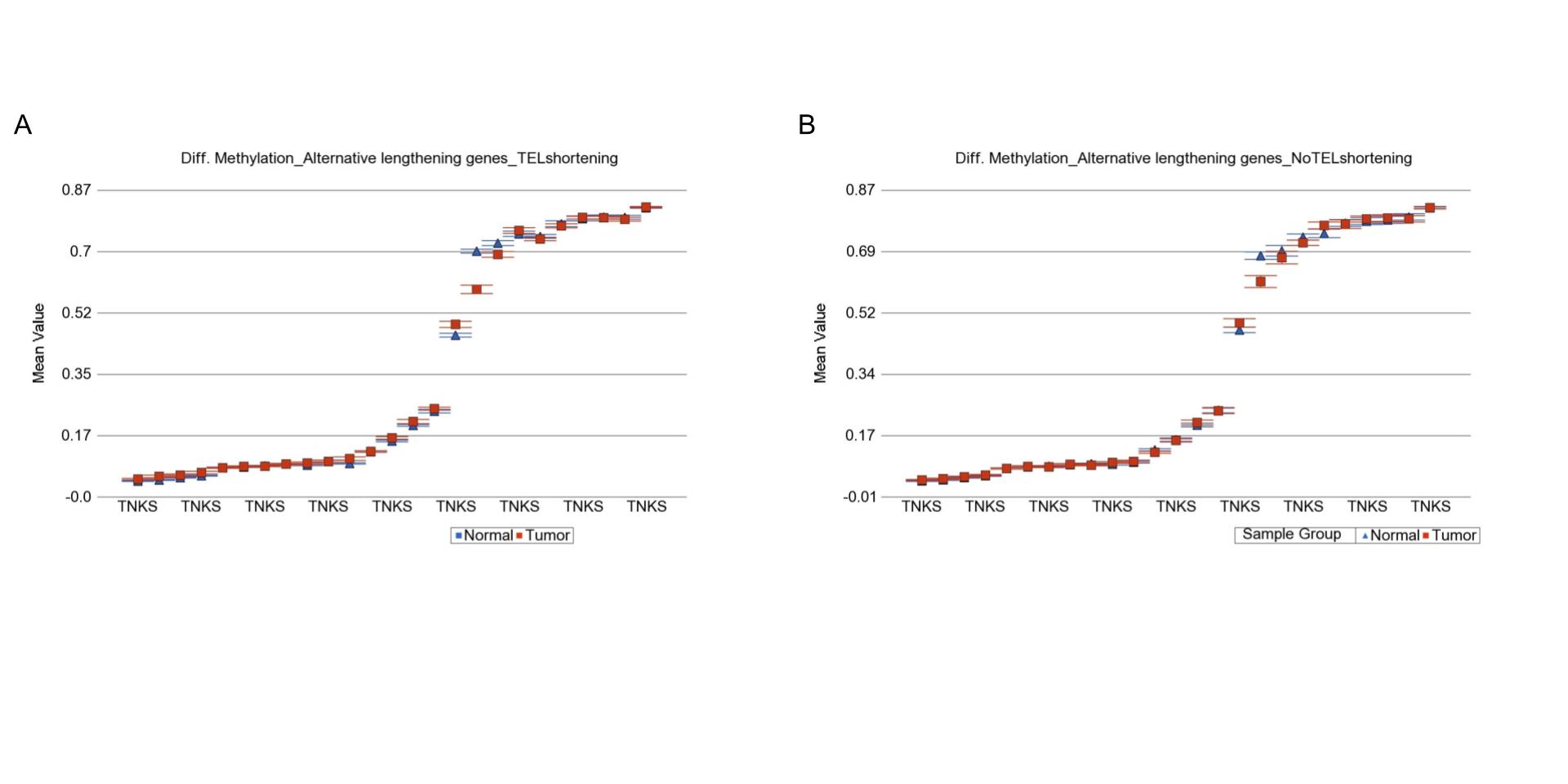

Supplement: Supplementary file 1 [file cancers-14-02250-s001.zip › Figure S8.jpg]

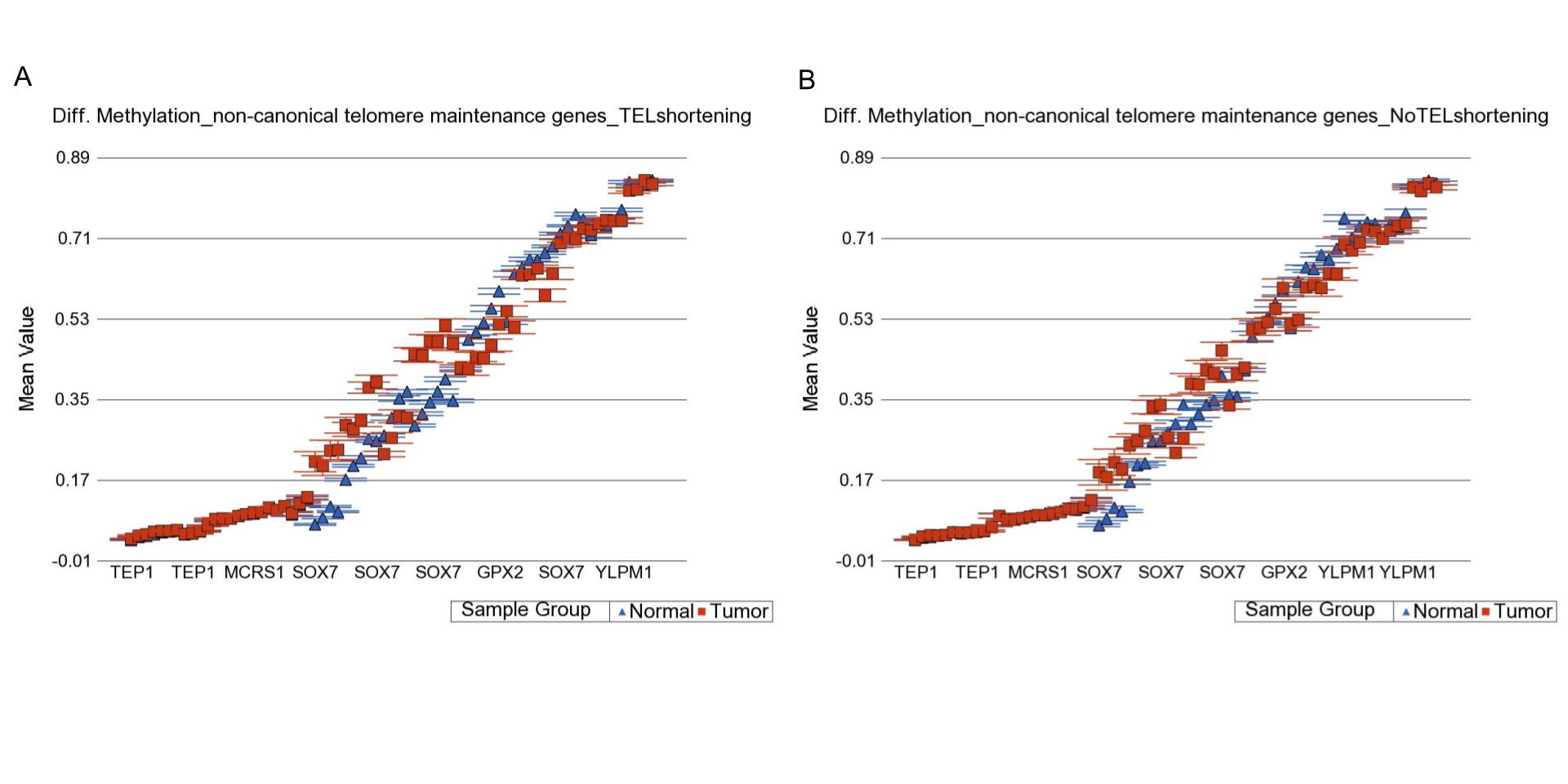

Supplement: Supplementary file 1 [file cancers-14-02250-s001.zip › Figure S9.jpg]
